# Association of Sport Participation and Calcium Intake with Bone Mineral Density in Children and Adolescents: A Cross-Sectional Study

**DOI:** 10.3390/children13030375

**Published:** 2026-03-06

**Authors:** Carla Caffarelli, Caterina Mondillo, Guido Cavati, Alessandro Versienti, Anna Lora, Sara Gonnelli, Stefano Gonnelli, Luigi Gennari, Antonella Al Refaie

**Affiliations:** 1 Department of Medicine, Surgery and Neuroscience, University of Siena, 53100 Siena, Italy; c.mondillo@student.unisi.it (C.M.); guido.cavati@student.unisi.it (G.C.); versienti2@student.unisi.it (A.V.); anna.lora@student.unisi.it (A.L.); saragonnelli@libero.it (S.G.); gonnelli@unisi.it (S.G.); luigi.gennari@unisi.it (L.G.); a.alrefaie@student.unisi.it (A.A.R.); 2 Department of Geriatrics, University Hospital of Nice, 06000 Nice, France; 3 Division of Internal Medicine I, San Giuseppe Hospital, 50053 Empoli, Italy

**Keywords:** bone mineral density, sport participation, calcium intake, fracture, children and adolescent

## Abstract

**Background**: Sport participation has been shown to have a positive impact on bone mineral density (BMD) in children and adolescents. In fact, the type, intensity, and duration of sports activities may influence the magnitude of the effect on BMD. The aim of this study was to examine the effect of different sports on BMD in children and adolescents. **Methods**: We studied 90 children and adolescents (age 10.21 ± 2.96 years): 43 soccer players, 27 vocational dancers, and 20 active controls. In all subjects, bone mineral density at the lumbar spine (BMD-LS), at the femoral neck (BMD-FN), and at the total femur (BMD-TH) was measured. Moreover, their daily dietary calcium intake was assessed, and the presence of prior fractures was reported. **Results**: The values of the BMD-LS Z-score adjusted for height did not differ between the three groups: controls (BMD-LS Z-score values were 0.41 ± 1.26, 0.16 ± 0.94, and −0.14 ± 0.96 for soccer players, vocational dancers, and active controls, respectively). On the contrary, BMD-FN and BMD-TH were significantly higher in the soccer players group compared to the vocational dancers and active controls groups (BMD-FN Z-score values, adjusted for height, were 0.49 ± 1.35, −0.04 ± 0.84, and −0.63 ± 1.25 for soccer players, vocational dancers, and active controls, respectively). Soccer players with a history of fractures showed no reduced BMD values compared to those without previous fractures, whereas vocational dancers and active controls with fracture history had reduced BMD values. **Conclusions**: Consistent athletic involvement during the pre-pubertal and pubertal years significantly enhances bone mineral acquisition. Specifically, youth soccer players demonstrate superior BMD at the proximal femur compared to less active peers, underscoring the site-specific osteogenic effect of sport-related mechanical strain.

## 1. Introduction

Bone mineral density (BMD) acquisition during childhood and adolescence is a critical determinant of lifelong skeletal health, influencing peak bone mass and reducing the risk of osteoporosis and fractures in adulthood [1,2]. The majority of adult bone mass is accrued during the first two decades of life, making this period crucial for optimizing bone development [3,4]. Suboptimal bone accretion during these formative years can lead to long-term skeletal fragility [4,5,6].

Skeletal development is a complex process influenced by a multitude of factors, including genetic predisposition, hormonal regulation, mechanical loading, and nutritional status [1,4,5,6,7]. Among the modifiable environmental factors, physical activity, particularly weight-bearing exercise, is widely recognized for its osteogenic potential [8,9]. Participation in various sports has been consistently linked to enhanced BMD in pediatric populations, with studies highlighting the beneficial effects of high-impact and multidirectional movements on bone remodeling [9,10,11]. However, the specific types, intensity, and duration of sport activities that optimize BMD in children and adolescents warrant further investigation.

Concurrently, adequate dietary calcium intake is indispensable for robust bone mineralization [12]. Calcium is the primary structural component of bone, and insufficient intake during periods of rapid growth can compromise bone density and increase fracture risk [12]. Dietary guidelines typically recommend specific daily calcium intakes for children and adolescents to support optimal skeletal accrual [13]. Despite these recommendations, concerns persist regarding whether children and adolescents consistently meet their calcium requirements, especially given evolving dietary patterns [13].

While the individual contributions of sport participation and calcium intake to bone health are well-established, their interactive effects in children and adolescents are less comprehensively understood. This study investigates the effects of various sports activities and dietary calcium intake on bone mineral density in a cohort of children and adolescents, using dual-energy X-ray absorptiometry (DXA).

## 2. Materials and Methods

### 2.1. Study Population

This non-profit, cross-sectional observational study involved 105 subjects, of both sexes, aged between 8 and 17 years (mean age 10.21 ± 2.96 years). These subjects were referred by General Practitioners and Primary Care Pediatricians to the Bone Densitometry outpatient clinics at the University Hospital of Siena (Italy) from January to December 2024. All participants provided written informed consent. The study was approved by the Institutional Review Board of Siena University Hospital (ID-23190/22; approved on 21 November 2022). The study protocol was explained in detail to all participants and their legal guardians. Subsequently, formal written informed consent was obtained from the parents, while subject assent was documented for the individual participants, ensuring full compliance with ethical guidelines. All data were anonymized prior to statistical analysis.

The study included individuals of both sexes, ranging from 8 to 17 years of age, who were referred for bone health evaluation. Patients with other medical conditions potentially affecting skeletal health or those on medications known to interfere with bone metabolism (such as heparin, thyroid hormones, antiepileptics, and corticosteroids) were excluded from the study. Additionally, patients receiving active vitamin D metabolites were also excluded.

Of the 105 patients screened, 6 were excluded for failing to meet the inclusion criteria or declining to participate, and an additional 7 subjects were excluded due to poor densitometric scan quality. The final analysis included 90 participants.

To evaluate the influence of sports activity on BMD, the included subjects were stratified into three distinct groups based on the type of activity practiced:

(1) **Soccer Player Group:** This group comprised 43 subjects who had been playing team soccer for at least three years, in addition to their school physical activity. Their training regimen included four hours per week, supplemented by one soccer match.
(2) **Vocational Dancer Group:** This group consisted of 27 subjects who had been enrolled in dance schools for at least three years, with a weekly commitment of at least three hours, in addition to school physical education.
(3) **Control Group:** This group included twenty subjects who participated exclusively in school physical activity.

**Figure 1:**
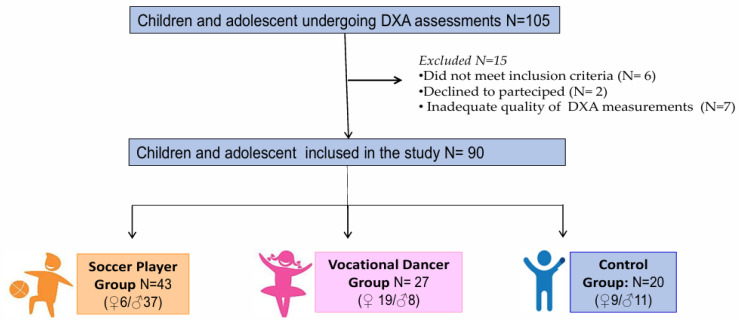
Flow chart showing the distribution of participants to the study.

The flow chart showing the distribution of participants in the study is presented in Figure 1.

### 2.2. Anthropometric Parameters

For all subjects, auxological parameters, birth weight, and the presence of previous fractures (spontaneous or due to minimal trauma) were assessed. Furthermore, data on pubertal development were collected. Weight was measured with scales (0.05 kg), and height was measured with a wall stadiometer (0.5 cm).

### 2.3. Dietary Calcium Assessment

Daily calcium intake was evaluated using a Food Frequency Questionnaire (FFQ). Moreover, participants adhered to their usual dietary patterns and did not use any dietary supplements. This 15-chapter questionnaire was developed based on data from the Italian National Institute of Nutrition regarding the composition of the Italian diet, food consumption frequency, and the relative importance of foods as calcium sources. This FFQ was validated in a large male and female population from the Siena area by comparing its results with those obtained from 14-day food diaries kept by the same subjects [14,15].

### 2.4. Dual-Energy X-Ray Absorptiometry Measurements

For all subjects, we performed BMD measurements at the lumbar spine (BMD-LS) and at femoral subregions (femoral neck: BMD-FN and total hip: BMD-T) using DXA (Discovery; Hologic, Inc., Bedford, MA, USA). All scans were performed by the same operator while the subjects were wearing light indoor clothing and no removable metal objects. The study involved healthy children who generally showed excellent cooperation; indeed, the majority maintained the required position autonomously. To guarantee data consistency, a specific pediatric protocol—including radiolucent aids and behavioral distraction—was available and implemented only in the few cases where movement risk was identified, ensuring adherence to the ALARA principle [16,17]. BMD Z-scores represent age, sex, and equipment-specific normative data for Caucasian children. According to the International Society for Clinical Densitometry (ISCD) official positions, a Z-score ≤ −2.0 is classified as “low bone mass for chronological age” [18,19]. To minimize the confounding effect of stature on bone mineral density (BMD) measurements, BMD Z-scores were adjusted for height-for-age Z-scores (HAZ) according to the methodology proposed by Zemel et al. [20]. This height-adjustment is essential to prevent the underestimation of BMD in subjects with short stature and its overestimation in taller individuals, providing a more accurate assessment of bone health relative to body size.

### 2.5. Statistical Analysis

The descriptive analysis of the study population and of the study parameters was carried out by considering mean and standard deviation (SD) or frequency. Clinical data and measured variable values across the study groups were compared using Student’s *t*-test for independent samples when normally distributed. For data not normally distributed, the non-parametric Kruskal–Wallis procedure was used to test the hypothesis that the means of quantitative variables were not significantly different across the different groups, while the extended Mantel–Haenszel chi-squared test was used to test for trend in proportions. For comparing two or more percentages or proportions, the chi-squared test was employed. All statistical analyses were performed using SPSS 10.1 software.

## 3. Results

### 3.1. Clinical Characteristics

Table 1 presents the clinical and laboratory characteristics of 90 subjects categorized by the type of physical activity performed. No significant differences were observed in weight, height, height Z-score, or age among the three groups, while a significant difference was found in birth weight values. Regarding lumbar spine BMD values, no significant differences were observed among the groups. Conversely, analysis of proximal femur BMD values revealed that the soccer player group had significantly higher values (*p* < 0.05) compared to both the dancer group and the healthy control group. Furthermore, the study population was stratified by sex; these findings are presented in the Supplementary Materials (Tables S1 and S2).

### 3.2. Dual-Energy X-Ray Absorptiometry

**Figure 2:**
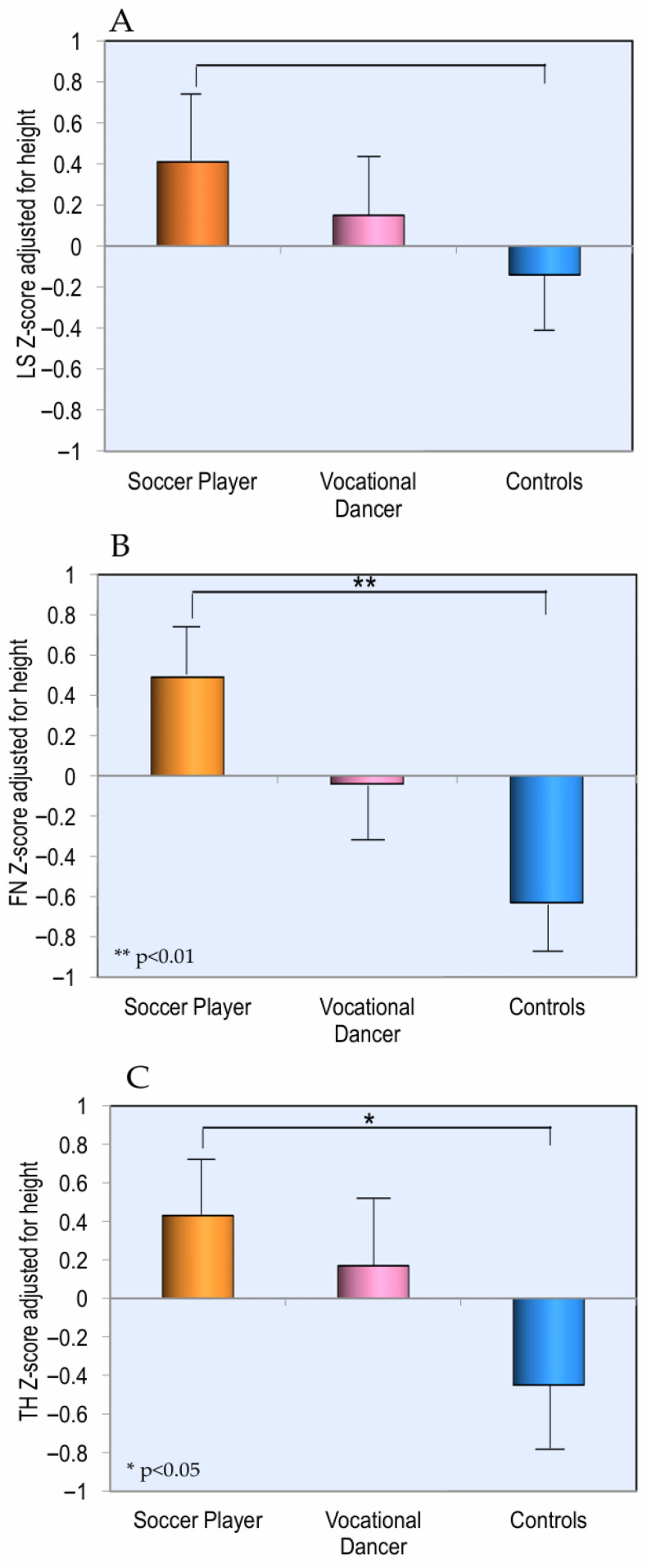
Values of BMD expressed as Z-score adjusted for height at lumbar spine (**A**), at femoral neck (**B**), and at total hip (**C**), grouped by different sports.

Additionally, as illustrated in Figure 2, lumbar spine BMD Z-score values showed no statistically significant differences among the three groups analyzed. As expected, femoral neck and total hip BMD Z-score values were significantly higher in the soccer player group compared to both the dancers and, notably, the healthy controls.

### 3.3. Calcium Intake

**Figure 3:**
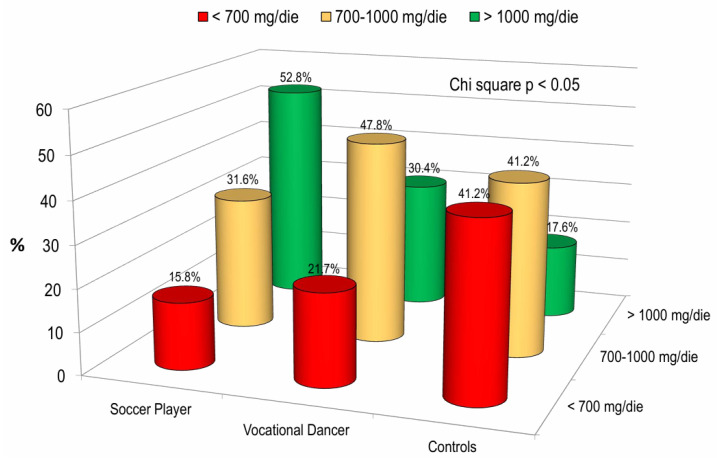
Dietary calcium intake within the study population stratified by sport.

Figure 3 illustrates the percentages of subjects with calcium intake < 700 mg/day, between 700 and 1000 mg/day, and >1000 mg/day, categorized by the type of sport performed. It is evident that soccer players have a significantly higher calcium intake compared to both dancers and healthy controls (*p* < 0.05).

### 3.4. Fractures

**Figure 4:**
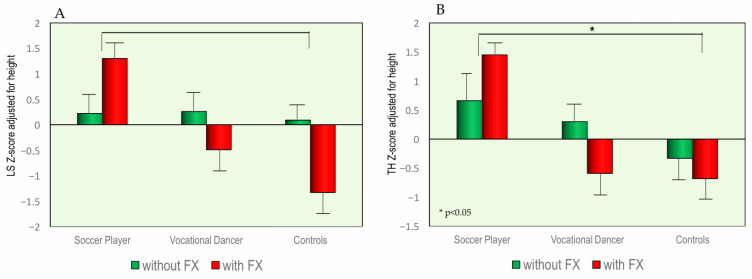
Values of BMD expressed as Z-score adjusted for height at lumbar spine (**A**) and at total hip (**B**) with and without a history of fracture, grouped by different sports.

Specifically, a total of 10 soccer players (13.3%) reported a fracture. The anatomical distribution of fractures in this group was as follows: three subjects had fractures of the radius or ulna, one subject reported tibia and fibula fractures, and six subjects presented with phalangeal fractures. Among the dancers, four subjects (5.3%) reported fractures. The anatomical locations of these fractures were as follows: one case of radius and ulna fracture, one clavicle fracture, one coccyx fracture, and one phalangeal fracture. Finally, in the control group, only two healthy subjects (2.7%) reported a history of previous fracture: one radius and ulna fracture and one humerus fracture. Figure 4 illustrates BMD Z-score values measured at the lumbar spine (LS) and total hip (TH), stratified by the presence or absence of a history of fractures. Analysis of the data for the soccer player group revealed no significant differences in BMD Z-score values between subjects with and without a history of previous fractures, both at the lumbar spine and proximal femur. In contrast, in the dancer and healthy control groups, subjects with a positive fracture history showed significantly lower BMD Z-score values compared to those without fractures, both at the lumbar spine and proximal femur.

## 4. Discussion

The results of the present study provide a comprehensive overview of how different types of physical loading and nutritional habits influence bone mass accrual and fracture risk in young populations. The primary finding—that soccer players exhibit significantly higher BMD at the proximal femur compared to dancers and sedentary controls—underscores the importance of the quality of mechanical loading over mere physical activity volume [21].

The disparity observed between the lumbar spine and the femoral neck/total hip BMD is particularly significant. While the lumbar spine, predominantly composed of trabecular bone, showed no significant differences among groups, the proximal femur, which contains a higher proportion of cortical bone and which responds differently to mechanical loading, exhibited higher bone mineral density values in the soccer player group compared to the control group. This site-specific adaptation is consistent with the “Mechanostat” theory proposed by Frost, which suggests that bone distribution is optimized in response to the highest local strains [22,23]. Soccer involves “high-impact” loading characterized by rapid accelerations, decelerations, and multi-directional changes in magnitude. These actions generate high ground reaction forces (GRFs) that primarily affect the weight-bearing sites of the lower limbs. Conversely, while dance involves weight-bearing, it often emphasizes repetitive movements and isometric control, which may not reach the same osteogenic threshold as the explosive movements found in field sports. In alignment with our study, previous cross-sectional and longitudinal research has demonstrated that soccer participation stimulates osteogenic adaptations, thereby enhancing bone formation and increasing areal bone mineral density [24,25,26].

Moreover, our data revealed that soccer players had a significantly higher calcium intake (>1000 mg/day) compared to the other two groups. This finding is crucial, as mechanical loading and nutrition work synergistically: physical activity increases the sensitivity of bone tissue to nutrients, while adequate calcium and vitamin D levels are permissive for the mineralization triggered by mechanical stress. In particular, as suggested by Specker et al. [26] and further supported by Daly [27], physical activity serves as the primary osteogenic stimulus, but its effectiveness is contingent upon nutritional status. Specifically, adequate levels of calcium and vitamin D act as permissive factors; without them, the bone remodeling triggered by mechanical loading cannot culminate in effective mineralization [2,3]. Vatanparast H et al. [28] investigated the influence of protein intake on bone mass measures in young adults, considering the influence of calcium intake through adolescence. The results indicate that when calcium intake is adequate, protein intake has a beneficial effect on the bone mass of young adult females, but in the absence of sufficient calcium intake, it does not confer as much benefit to bone [29].

On the contrary, the lower calcium intake observed in dancers is a cause for clinical concern. In esthetic and lean-demand sports, athletes often face “Relative Energy Deficiency in Sport” (RED-S). The results observed in the dancer group appear to align with the clinical framework of RED-S. This becomes particularly critical in individuals competing in esthetic sports at an elite level, where the emphasis on physical appearance and leanness often leads to compromised nutritional status [30]. The resulting chronic energy deficit and micronutrient insufficiency can trigger hypogonadotropic hypogonadism, which impairs bone formation and accelerates resorption, potentially negating the osteogenic benefits typically derived from physical exercise [31]. This hormonal suppression disrupts the bone remodeling balance—favoring resorption over formation—and may neutralize the positive effects of mechanical loading on bone mineral density [32].

Consistent with previous findings [8,33], vocational dancers in our cohort exhibited lower BMD and BMD Z-scores. Furthermore, the significantly diminished Z-scores observed in dancers with a history of fractures suggest that skeletal integrity may be compromised by chronic energy deficiency. This systemic impairment likely increases susceptibility to injury, even during routine, non-traumatic physical activities [34].

A paradox appears in our results: the soccer group exhibited the highest BMD but also the highest percentage of fractures (13.3%). However, the anatomical distribution provides clarity. Most soccer-related fractures occurred in the phalanges or the forearm (radius/ulna), suggesting acute traumatic events typical of contact sports [35]. This distinguishes the soccer group from the dancers, whose injuries are more likely linked to bone quality degradation rather than external impact [36]. This is further supported by the fact that in the soccer group, there was no difference in BMD between those with and without fractures, indicating that these injuries were likely due to the force of the impact exceeding the capacity of even healthy bone [35,36]. In contrast, in the dancer and control groups, subjects with a history of fracture had significantly lower BMD Z-scores. This suggests that in these populations, a lower peak bone mass acts as a predisposing factor, leading to “low-energy” or fragility-related fractures during daily activities or routine training [37].

The significance of these findings extends beyond youth athletic performance, touching upon the fundamental concept of peak bone mass (PBM). The PBM achieved during adolescence and young adulthood is the primary determinant of the risk for osteoporosis and fragility fractures in later life [5]. The attainment of an optimal PBM during the first two decades of life is considered a primary strategy for preventing osteoporosis and fragility fractures in later life, specifically, a 10% increase in peak bone mass has been estimated to delay the clinical onset of osteoporosis by approximately 13 years [13]. Since bone mass begins to decline naturally after the third decade, the “skeletal bank account” built during adolescence determines how long an individual can remain above the clinical threshold for fractures [3,5]. Physical activity, particularly high-impact and multi-directional loading (such as jumping, running, or dancing), is the most potent non-pharmacological stimulus for bone formation. According to the mechanostat theory, bone adapts to the mechanical strains placed upon it by increasing osteoblastic activity [9,10,11,13]. Calcium is the fundamental structural component of the bone matrix. During the rapid growth phases of adolescence, the skeletal demand for calcium is at its peak. Without adequate calcium bioavailability, the bone cannot mineralize the new tissue created by exercise-induced stimulation [12,38].

The present study has several limitations. While this study benefits from a well-categorized cohort, the cross-sectional design prevents us from establishing a definitive causal relationship between specific drills and bone density. In particular, given the demographic distribution of the disciplines analyzed, the soccer sample is predominantly male, while the dance sample is mostly female. Failure to correlate the influence of gender with physical activity, bone density, and calcium intake would introduce significant bias into the conclusions. Furthermore, factors such as vitamin D serum levels and hormonal status (e.g., estrogen levels in female subjects) were not included in this analysis but likely play a role in the observed differences.

## 5. Conclusions

In conclusion, soccer participation during youth emerges as a potent protective factor for the improvement of BMD values, driven not only by high-intensity mechanical loads but also by better adherence to adequate nutritional regimens. Prevention programs should aim to integrate high-impact activities and close monitoring of calcium intake, especially in disciplines such as dance, where the risk of energy and micronutrient deficits is higher.

## Figures and Tables

**Table 1:** Anthropometric and Instrumental Characteristics of the Study Population.

Characteristics	Soccer Player Group	Vocational Dancer Group	Control Group	*p*
Age (years)	10.7 ± 2.8	10.3 ± 2.3	9.3 ± 3.7	-
Weight (kg)	39.9 ± 12.2	38.5 ± 17.3	34.5 ± 14.1	-
Height (cm)	146.3 ± 16.6	144.2 ± 16.5	138.1 ± 22.3	-
Height Z-score	0.25 ± 1.03	0.21 ± 0.88	−0.16 ± 2.32	-
Calcium Intake (mg/day)	925 ± 389	853 ± 289	774 ± 305	0.05
Birth weight (kg)	3.390 ± 0.262	3.520 ± 0.344	3.190 ± 0.562	0.05
BMD-LS (g/m^2^)	0.714 ± 0.188	0.692 ± 0.168	0.627 ± 0.150	-
BMD-FN (g/m^2^)	0.801 ± 0.209	0.694 ± 0.102	0.666 ± 0.124	0.05
BMD-TH (g/m^2^)	0.849 ± 0.228	0.762 ± 0.115	0.727 ± 0.127	0.05

## Data Availability

The data that support the findings of this study are available from the corresponding author (C.C.).

## Supplementary Materials

The following supporting information can be downloaded at https://www.mdpi.com/article/10.3390/children13030375/s1: Table S1: Anthropometric and Instrumental Characteristics of the Male Subjects of the Study Population; Table S2: Anthropometric and Instrumental Characteristics of the Female Subjects of the Study Population.

## Abbreviations

The following abbreviations are used in this manuscript:

BMD	Bone Mineral Density
BMD-LS	Bone Mineral Density at Lumbar Spine
BMD-FN	Bone Mineral Density at Femoral Neck
BMD-TH	Bone Mineral Density at Total Hip
DXA	Dual-Energy X-Ray Absorptiometry
FFQ	Food Frequency Questionnaire
GRFs	Ground Reaction Forces
