# Supplementary material for: Association of Sport Participation and Calcium Intake with Bone Mineral Density in Children and Adolescents: A Cross-Sectional Study
Source: Children (Basel). 2026 Mar 6;13(3):375. doi: 10.3390/children13030375 (PMC13025829; doi:10.3390/children13030375)
Supplement: Supplementary file 1 [file children-13-00375-s001.zip › children-4133994-supplementary.pdf]

Table S1: Anthropometric and Instrumental Characteristics of the Male Subjects of the Study Population

**Characteristics**	**Soccer Player Group**	**Vocational Dancer Group**	**Control Group**	**p**
N	37	8	9	
Age (years)	10.8 ± 2.8	9.6 ± 3.1	9.5 ± 4.5	-
Weight (Kg)	45.1 ± 12.3	43.8 ± 18.7	36.6 ± 15.9	-
Height (cm)	147.5 ± 16.9	145.4 ± 19.3	138.7 ± 23.5	-
Height Z-score	0.19 ± 1.01	0.37 ± 0.63	0.39 ± 1.07	-
BMD-LS (g/m^2)	0.718 ± 0.193	0.620 ± 0.138	0.593 ± 0.239	-
LS Z-score adjusted for height	0.42 ± 1.30	-0.12 ± 0.66	0.02 ± 1.08	-
BMD-FN (g/m^2)	0.806 ± 0.215	0.683 ± 0.102	0.686 ± 0.153	0.05
FN Z-score adjusted for height	0.47 ± 1.39	-0.30 ± 0.47	-0.69 ± 1.42	0.05
BMD-TH (g/m^2)	0.856 ± 0.233	0.712 ± 0.116	0.769 ± 0.160	-
FN Z-score adjusted for height	0.42 ± 1.43	-0.40 ± 0.74	-0.52 ± 1.18	0.05

Table S2: Anthropometric and Instrumental Characteristics of the Female Subjects of the Study Population

Characteristics	Soccer Player Group	Vocational Dancer Group	Control Group	P
N	6	19	11	
Age (years)	9.5 ± 1.7	10.1 ± 2.2	8.8 ± 3.2	-
Weight (Kg)	34.5 ± 7.7	36.8 ± 11.4	30.4 ± 12.3	-
Height (cm)	142.0 ± 11.3	143.5 ± 15.1	134.6 ± 18.6	-
Height Z-score	1.25 ± 1.12	0.26 ± 0.94	-0.73 ± 1.82	-
BMD-LS (g/m^2)	0.658 ± 0.78	0.714 ± 0.170	0.661 ± 0.156	-
LS Z-score adjusted for height	0.32 ± 0.16	0.26 ± 0.99	-0.32 ± 0.85	-
BMD-FN (g/m^2)	0.710 ± 0.109	0.702 ± 0.104	0.625 ± 0.098	-
FN Z-score adjusted for height	0.81 ± 0.34	0.04 ± 0.89	-0.67 ± 1.21	0.05
BMD-TH (g/m^2)	0.744 ± 0.105	0.774 ± 0.115	0.691 ± 0.087	-
FN Z-score adjusted for height	0.61 ± 0.11	0.32 ± 0.85	-0.38 ± 0.97	0.09
